# Association of G12D mutation in the KRAS gene with HPV and EBV in gastrointestinal cancer tissues

**DOI:** 10.1177/03000605241302302

**Published:** 2024-12-14

**Authors:** Vahideh Hamidi Sofiani, Arefeh Ebrahimian Shiadeh, Alijan Tabarraei, Hadi Razavi Nikoo, Farzin Sadeghi, Ghodsieh Kamrani, Yousef Yahyapour, Abdolvahab Moradi

**Affiliations:** 1 Department of Microbiology, 125691 Faculty of Medicine, Golestan University of Medical Sciences, Gorgan, Iran; 2 Department of Medical Microbiology, Faculty of Medicine, Babol University of Medical Sciences, Babol, Iran; 3 Department of Pathology, 114456 School of Medicine, Faculty of Medicine and Clinical Research Development Center, Babol University of Medical Sciences, Babol, Iran

**Keywords:** Gastric cancer, colorectal cancer, KRAS G12D, human papillomavirus, Epstein–Barr virus, mutation

## Abstract

**Objective:**

This study aimed to explore the potential relationship between viral infections and gastrointestinal (GI) malignancies, focusing on the presence of KRAS G12D mutations. Specifically, we investigated the association of viral agents, including human papillomavirus (HPV) and Epstein–Barr virus (EBV), with KRAS G12D mutations in GI cancers to better understand their combined role in cancer development.

**Methods:**

This cross-sectional study comprised 92 patients diagnosed with GI cancer and 100 healthy individuals in the control group. All samples were examined to detect the KRAS G12D gene mutation and the existence of HPV and EBV using real-time polymerase chain reaction assays.

**Results:**

HPV and EBV DNA were detected in 5.4% and 51.4% of gastric cancer samples and in 7.3% and 49.1% of colorectal cancer samples, respectively. Analysis of KRAS G12D in plasma samples revealed heterozygous mutations in 54% of patients with gastric cancer and 35% of patients with colorectal tumors. Among EBV-positive colorectal cancer samples, 1.8% were wild-type, while 47.2% exhibited heterozygous mutations. Among HPV-positive colorectal cancer patients, 1.8% exhibited wild-type KRAS, 5.4% had heterozygous mutations, and 3.2% had homozygous mutations.

**Conclusion:**

This study detected a significant correlation between the presence of viral agents and KRAS G12D mutations.

## Introduction

Gastrointestinal (GI) tract malignancies are among the most common types of cancer worldwide. Colorectal cancer and gastric cancer rank third and fourth in global cancer prevalence. However, they both stand as the second most common causes of cancer-related mortality worldwide.^1^ Currently, approximately 20% of cancer cases are attributed to infectious agents, such as viral infections.^2^ Human papillomaviruses (HPVs), for instance, have been linked to multiple types of mucoepithelial cancer, including cervical (99.7%), oropharyngeal (52%), penile (65%), vulvar (62%), vaginal (84.25%), and anal cancer (80%).^3,4^ Furthermore, research has explored potential involvement of these viruses in some types of cancer, such as gastric and colorectal cancer. The perception is that the presence of HPV in colorectal cancer may stem from contamination or secondary infection rather than being the direct cause of the disease. Epstein–Barr virus (EBV) is another viral agent associated with the development of numerous malignancies, including Burkitt’s lymphoma, nasopharyngeal carcinoma, and gastric cancer.^5^

*Kristen rat sarcoma viral oncogene homolog* (KRAS (HGNC ID: 6407)) is a gene that plays a significant role in cell signaling and is frequently mutated in various tumors, particularly colorectal cancer. Although there is no direct correlation between HPV and KRAS mutations, both can independently contribute to the development of different types of cancer. In certain instances, HPV infections may influence cell behavior and potentially interact with other genetic factors, such as KRAS mutations, possibly promoting cancer development. It is important to note that the relationships between these factors can differ depending on the specific cancer type and individual cases. Furthermore, there is no definitive answer regarding the direct effect of EBV on KRAS mutations.^6^

We examined KRAS mutations in the context of HPV and EBV infection to clarify the potential correlations between viral infection and genetic changes that occur within cells. Previous studies have explored the potential for viruses such as HPV and EBV to interact with genetic mutations such as those in the KRAS gene to determine whether synergistic mutations exist that contribute to the initiation or progression of cancer.^7^ However, the specific relationship between these viruses and KRAS remains an area of ongoing research characterized by complex and multifaceted mechanisms. The objective of the current study is to determine how these mutations interact with viral infections and affect disease outcomes, thereby potentially offering valuable insights into the underlying mechanisms that drive disease progression and therapeutic strategies. In this context, we investigated circulating tumor DNA (ctDNA)/KRAS in conjunction with HPV and EBV infections in gastric and colorectal cancer.

## Materials and methods

### Patients

This cross-sectional study included patients diagnosed with histologically confirmed colorectal cancer and gastric cancer. Tissue samples were obtained from the Golestan Population-based Cancer Registry's biobank in October 2022. Of note, normal adjacent tissue (NAT) collected from these patients was used as the control group. During the period from August to December 2022, patients diagnosed with gastric and colorectal cancer were recruited from Ayatollah Rouhani Hospital and the Omid Clinic in Babol. The samples comprised biopsy specimens, surgical resections, and formalin-fixed paraffin-embedded blocks. The inclusion criteria consisted of a singular cancer type diagnosis confirmed by experienced pathologists and the ability of patients to provide written informed consent to participate in the study. The exclusion criteria included patients with significant uncontrolled medical conditions such as severe cardiovascular disease, active infections (e.g., human immunodeficiency virus, hepatitis), or organ failure (e.g., liver or kidney failure). Patients without demographic information were excluded from the study. In addition, results that fell outside the scope of the study were excluded. The study was conducted in accordance with the principles of the Declaration of Helsinki and was approved by the Ethics Committee of the Golestan University of Medical Sciences (Ethics Code: IR.GOUMS.REC.1401.134). All participants provided written informed consent and received comprehensive information regarding the study's procedures and objectives. Additionally, all patient details have been de-identified. The reporting of this study conforms to the STROBE guidelines.^8^

### KRAS sample preparation

Colorectal and gastric cancer tissue, NAT, and blood samples were collected from patients, and blood samples from healthy individuals (control group) were randomly collected. Tissue and blood samples were analyzed to investigate KRAS mutations in colorectal and gastric cancer patients, as well as in healthy individuals.

### Polymerase chain reaction (PCR) for screening KRAS mutations (exon 2)

DNA extracted from collected tissues was investigated for KRAS mutations, and ctDNA was extracted from plasma samples using the standard phenol-chloroform method for DNA extraction. Mutation analysis of KRAS codon 12 (G12D) was conducted using G12D KRAS and wild-type KRAS forward and reverse primers for real-time PCR amplification.^9^ Amplification reactions were carried out using 12.5 µL SybrGreen PCR master mix (Amplicon 2X, QIAGEN, GmbH, Hilden, Germany), 1 µL of forward and reverse primers (25 pm), 5 µL (100 ng) of extracted DNA and ctDNA, and 5.5 µL of distilled water in a final volume of 25 µL. The PCR program is displayed in Table 1.

**Table 1.** Primer sequences of the KRAS and polymerase chain reaction (PCR) program.

Type of primer		Primer sequence	Amplicon size (bp)	Ref
Wild type	Forward	5′-TGTGGTAGTTGGAGCTGG-3′	175	[9]
Mutant	Forward	5′-TGTGGTAGTTGGAGCTGA-3′		
Reverse		5′-TCATGAAAATGGTCAGAGAAACC-3′		
PCR Program		Initial denaturation steps at 95°C for 15 min, followed by 35 cycles at 95°C for 30 s, 59°C for 30 s, and 72°C for 30 s.		

### HPV screening

The collected tissue samples were investigated to detect HPV. Viral genome extraction using the DNA FFPE Tissue Kit (PZP, Tehran, Iran) was conducted according to the manufacturer’s instructions for using the GAPDH gene as a housekeeping gene to assess the extracted nucleic acids. Additionally, a tissue DNA extraction kit (Sinaclon, Tehran, Iran) was employed to extract the viral genome from fresh tissue according to the manufacturer’s instructions. Purified DNA was kept at −20°C until use. HPV DNA was identified using the real-time PCR method. The HPV profile was determined using GP5+/GP6+ consensus primers for the L1 region (150 bp product) (Table 2).^9^ Amplification reactions were carried out using 12.5 µL of SybrGreen PCR master mix (Amplicon 2X), 0.5 µL of GP5+/6+ primers (50 pm), 4 µL (100 ng) of extracted DNA, and 7.5 µL of distilled water in a final volume of 25 µL. The reactions were conducted using an Applied Biosystems Step One Plus Real-Time PCR System from Thermo Fisher Scientific (Waltham, MA, USA). The PCR program is given in Table 2. DNA extracted from the HeLa cell line was incorporated as a positive control in each set of real-time PCR assays.

**Table 2.** Primer sequences for human papillomavirus.

Primer name	Primer sequence	Ref
GP5+:	5′-TTTGTTACTGTGGTAGATACTAC-3′	[51]
GP6+:	5′-GAAAAATAAACTGTAAATCATATTC-3′	
PCR Program	Initial denaturation step at 95°C for 10 minutes, followed by 40 cycles at 95°C for 15 s, 55°C for 50 s, and 72°C for 1 minute.	

### EBV screening

The collected samples were analyzed to detect the presence of EBV. The detection and quantification of the EBV viral load in the extracted samples was conducted through quantitative real-time PCR using a Rotor-Gene Q real-time PCR system (QIAGEN). This was accomplished using two primer sets and a TaqMan probe specific for the EBV BALF5 and EBER genes. The EBV viral load was calculated as the ratio of viral DNA copies to half of the RNase P gene copy (each diploid cell contains two copies of the RNase P gene), allowing for the normalization of viral copies to the number of cell equivalents, as previously described by Teresa et al.^10^ The plasmids containing cloned target sequences of EBV BALF5, EBER, and the human RNase P gene (quantitative standards for real-time PCR) were constructed by a gene synthesis service (Shanghai Gene Ray Biotech Co., Ltd., Shanghai, China). Each amplification reaction was carried out using 100 ng of extracted DNA, 12.5 µL of qPCR Probe Master Mix without ROX (Amplicon, Odense, Denmark), each primer (0.3 µM), and a dual-labeled probe (0.2 µM) in a total reaction volume of 25 µL. DNA extracted from the supernatant of an EBV-producing B-cell line (B95-8) was included as a positive control in each real-time PCR set. The PCR program is provided in Table 3.

**Table 3.** Primer sequences for Epstein–Barr virus (EBV).

Target gene	Primer and probe	Sequence (5′-3′)	PCR Program	Ref
Human RNase P	RNP F-PrimerRNP R-PrimerRNP Probe	5′-AGATTTGGACCTGCGAGCG-3′5′-GAGCGGCTGTCTCCACAAGT-3′FAM-TTCTGACCTGAAGGCTCTGCGCG-BHQ1	Initial denaturation step at 95°C for 15 minutes, followed by 45 cycles at 95°C for 15 s and 60°C for 20 s	[52]
EBV EBER	EBER-F-PrimerEBER-R-PrimerEBER-Probe	5′-TGACGTAGTCTGTCTTGAGGAGATG-3′5′-CGTCTCCTCCCTAGCAAAACC-3′FAM-TGCAAAACCTCAGGACCTACGCTGC-TAMRA	Initial denaturation step at 95°C for 15 minutes, followed by 45 cycles at 95°C for 15 s and 60°C for 20 s	[53]
EBV-BALF5	BALF5-F-PrimerBALF5-R-PrimerBALF5-Probe	5′-CGGAAGCCCTCTGGACTTC-3′5′-CCCTGTTTATCCGATGGAATG-3′FAM-TGTACACGCACGAGAAATGCGCC-BHQ1	Initial denaturation step at 95°C for 15 minutes, followed by 45 cycles at 95°C for 15 s and 62°C for 60 s	[54]

### Statistical analysis

All statistical analyses were conducted using IBM SPSS version 27 software (IBM Corp., Armonk, NY, USA). Patient data comparisons were performed with statistical significance defined as p < 0.05. The chi-square test and Fisher’s exact test were used to evaluate significant differences, with p values less than 0.05 considered statistically significant. To ensure the genetic variation observed in the population was consistent with expected distributions, all results were assessed according to the Hardy–Weinberg equilibrium, which helps to verify the absence of significant genotypic deviations in the sample population.

## Results

### Patient characteristics

The study consisted of 92 patients (a total of 184 tissues, including 55 colorectal cancer tissue samples, 55 colorectal NAT samples along with blood samples, 37 gastric cancer tissue samples, and 37 gastric NAT samples along with blood samples, with 53 collected from the Golestan Population-based Cancer Registry's biobank and 39 collected from Ayatollah Rouhani Hospital and the Omid Clinic in Babol) with histologically confirmed GI cancer, including 55 men and 37 women, with a mean age of 57 ± 1.2 years. In addition, blood samples were randomly collected from a control group of 100 healthy individuals, consisting of 55 men and 45 women, with a mean age of 51.3 ± 1.4 years, for comparison. The study concentrated on the analysis of ctDNA for colorectal and gastric cancer, with the objective of comparing the status of exon 2 of the RAS gene between plasma and tissue samples.

Among the mutations observed in Ras gene exon2, the G12D mutation was prevalent in both gastric and colorectal cancer patients. The mutation status was further categorized as homozygous or heterozygous. Detailed experimental results are shown in Table 4, which reveals a significant correlation between the cancer and control groups in terms of KRAS status according to Fisher's exact test (p < 0.05). In addition, investigation of KRAS mutations in gastric and colorectal cancer revealed a p value of 0.38. Moreover, the analysis of KRAS mutations in gastric control and colorectal control NAT samples showed a p value of 0.08.

**Table 4.** KRAS analysis in cancerous and normal tissues.

Type of sample	Wild type	Mutant heterozygote	Mutant homozygote	Total	p values
Gastric cancer	4 (11%)	32 (86%)	1 (3%)	37	p = 0.009
Gastric control(Normal tissue adjacent to the tumor)	0 (0%)	32 (82%)	7 (18%)	39	
Colorectal cancer	4 (7 %)	51 (93%)	0 (0%)	55	p = 0.02
Colorectal control(Normal tissue adjacent to the tumor)	1 (2%)	53 (95%)	2 (3%)	56	

In colorectal cancer, a substantial majority of patients, 93%, exhibited heterozygous mutations. In NAT within colorectal samples, 95% of tissues displayed heterozygous mutations, and 3% exhibited homozygous mutations (p = 0.02). However, there was a difference in the mutation pattern observed in gastric cancer. Specifically, 86% of cases exhibited heterozygous mutations, while 3% demonstrated homozygous mutations. In the case of NAT in gastric samples, 82% of tissues showed heterozygous mutations, while 18% exhibited homozygous mutations (p = 0.009). Analysis of plasma samples from gastric cancer patients showed that 54% had heterozygous mutations in the RAS gene, while 46% had the wild-type gene. In comparison, in the gastric cancer patient control group, 33% had heterozygous mutations and 67% had the wild-type gene (p = 0.01).

According to the chi-square and Fisher’s exact tests (Table 5), no significant association of age and sex with colorectal cancer was found. However, a significant correlation was identified between gastric cancer and patient age (p < 0.001). According to Fisher's exact test (Table 6), no significant correlation was found between the KRAS status in colorectal and gastric cancer and the histological characteristics of the cancers. Moreover, a significant relationship (p < 0.05) was identified between the KRAS gene status in gastric and colorectal cancer and that of the control group in tissue samples. Additionally, the KRAS gene status in ctDNA was examined in control plasma samples (Table 7). The plasma samples of the two control groups demonstrated a significant correlation (p < 0.05). The detailed results are presented in Table 7.

**Table 5.** Demographic and pathological characteristics of patients.

Characteristics	Sex		Age (years)							Carcinoma grade*		
	Male	Female	<30	30–40	40–50	50–60	60–70	70–80	>80	Well	Moderate	Poor
Colorectal cancer	31	24	1	2	6	18	14	10	4	21 (47.7%)	20 (45.5%)	3 (6.8%)
Colorectal control	32	18	2	6	10	14	12	5	1			
p value	p = 0.851		p = 0.38									
Gastric cancer	24	13	0	2	2	4	13	13	3	10 (40%)	3 (12%)	12 (48%)
Gastric control	22	26	6	6	8	13	13	2	0			
p value	p = 0.066		p < 0.001									

*For colorectal and gastric cancer, the pathological characteristics of 11 and 12 patients, respectively, could not be assessed because of the small size of biopsy samples.

**Table 6.** Investigation of the association of KRAS status with pathological characteristics of patients.

Characteristics (Carcinoma grade)*	Colorectal cancer		Gastric cancer		
	KRAS Status		KRAS status		
	Wild type	Heterozygote	Wild type	Heterozygote	Homozygote
Well	2	19	1	9	0
Moderate	1	19	0	3	0
Poor	1	2	3	8	1
p value	p = 0.334		p = 0.799		

*For colorectal and gastric cancer, the pathological characteristics of 11 and 12 patients, respectively, could not be assessed because of the small size of biopsy samples.

**Table 7.** KRAS analysis in plasma specimens.

Types of samples	Wild type	Mutant heterozygote	Total	p values
Gastric control	32 (67%)	16 (33%)	48	p = 0.01
Colorectal control	44 (88%)	6 (12%)	50	

Within the context of colorectal cancer, 35% of patients displayed heterozygous mutations, whereas the remaining 65% demonstrated the wild-type gene. However, in the colorectal control group, 88% had the wild-type gene and 12% had heterozygous mutations in plasma (p < 0.001).

### HPV and EBV status in gastric and colorectal cancer

All samples were analyzed for the presence of HPV and EBV. Within the gastric cancer group, 2 of 37 samples were HPV-positive, 19 of 37 were EBV-positive, and 2 exhibited coinfections with HPV and EBV. In the colorectal cancer group, 4 samples were HPV-positive, 27 of 55 samples were EBV-positive, and 1 coinfection with HPV and EBV were found. Among gastric NAT samples, 5 of 37 samples were EBV-positive, and none of the samples in the gastric cancer group were positive for HPV. Among colorectal NAT samples, 9 of 55 were EBV-positive, and 2 were HPV-positive (Table 8).

**Table 8.** Prevalence of human papillomavirus (HPV) and Epstein-Barr virus (EBV) positivity in tissue samples.

Type of sample	HPV positive	EBV positive	HPV/EBV positive	Total
Gastric cancer	2 (5.4%)	19 (51.4%)	2 (5.4%)	37
Gastric control	0 (0.0%)	5 (13.5%)	0 (0.0%)	37
p value	p = 0.222	p < 0.001	p = 0.222	
Colorectal cancer	4 (7.3%)	27 (49.1%)	1 (1.9%)	55
Colorectal control	2 (3.6%)	9 (16.4%)	2 (3.6%)	55
p value	p = 0.421	p < 0.001	p = 1	

**Figure 1.**
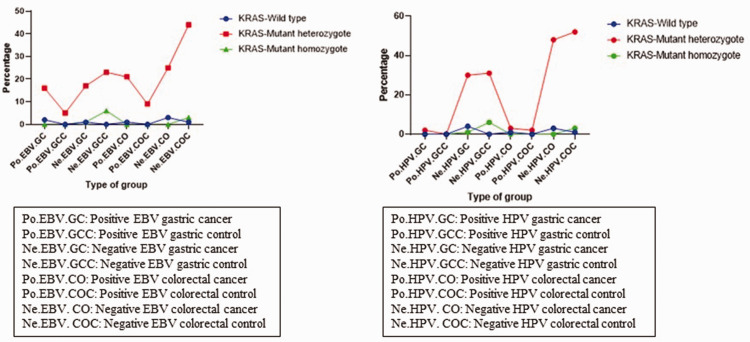
KRAS mutation profiles in samples Infected with human papillomavirus (HPV) and Epstein–Barr virus (EBV).

According to the chi-square and Fisher's exact test (Table 8), no significant association was found between HPV and colorectal and gastric cancer. However, a significant correlation was identified between EBV and gastric and colorectal cancer (p < 0.001). As shown in Figure 1, samples without HPV or EBV infection showed a high KRAS heterozygote mutation rate in colorectal and gastric cancer patients.

Separate analyses of tissue and plasma samples were conducted to detect KRAS mutations. The status of this gene was compared between tissue and plasma samples, and the RAS gene was characterized into four distinct categories: tissue and plasma positive for KRAS mutation, tissue and plasma negative for KRAS mutation, tissue negative but plasma positive for KRAS mutation, and tissue positive but plasma negative for KRAS mutation (Table 9). Additionally, we analyzed the control samples to investigate an association between HPV and EBV infection and KRAS mutations (Table 10). It was expected that the mutation status of KRAS in cancerous tissue would be the same as that in plasma. According to the results, this condition was observed in most gastric and colorectal samples. The reason for the difference in the remaining samples could be related to factors such as the cancer stage.

**Table 9.** KRAS situation with HPV and EBV infection.

KRAS status in tissue and plasma	Stomach cancer						Colorectal cancer					
	HPV		EBV		HPV/EBV		HPV		EBV		HPV/EBV	
	Positive	Negative	Positive	Negative	Positive	Negative	Positive	Negative	Positive	Negative	Positive	Negative
Tissue and plasma positive KRAS mutation	0	17	12	6	0	18	0	23	9	14	0	23
Tissue and plasma negative KRAS mutation	0	1	0	0	0	0	1	1	1	1	1	1
tissue negative plasma positive KRAS mutation	0	3	2	1	0	3	0	2	0	2	0	2
Tissue positive plasma negative KRAS mutation	2	14	5	11	2	14	3	24	17	10	0	26
Total	2	35	19	18	2	35	4	50	27	27	2	53

**Table 10.** KRAS situation with HPV and EBV infection in control groups.

KRAS situation in tissue	Stomach control						Colorectal control					
	HPV		EBV		HPV/EBV		HPV		EBV		HPV/EBV	
	Positive	Negative	Positive	Negative	Positive	Negative	Positive	Negative	Positive	Negative	Positive	Negative
Wild type	0	0	0	0	0	0	0	1	0	1	0	1
Mutant heterozygote	0	31	6	25	0	31	2	52	9	45	2	52
Mutant homozygote	0	6	0	6	0	6	0	3	0	3	0	3

**Figure 2.**
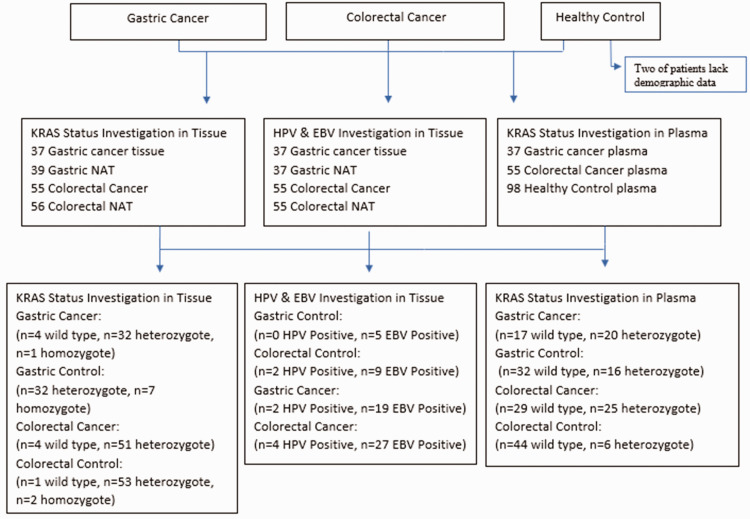
Flow chart showing patient selection and classification in a cross-sectional study that evaluated the relationship between plasma and tissue samples.

As shown in Table 10, the KRAS gene pattern in most gastric and colorectal cancer tissues was that of a heterozygous mutation. This finding indicates that in the majority of patients, KRAS mutation occurs in only one allele. Moreover, the mutation pattern did not change despite viral infection, and in cases of HPV and EBV positivity, heterozygous mutations were still observed (Figure 2).

## Discussion

The presence of *KRAS* mutations has been linked to reduced response rates to specific chemotherapeutic agents. Therefore, it is essential to ascertain the KRAS mutational status when contemplating the use of targeted therapies. A link between KRAS gene mutations and the therapeutic response was initially observed in individuals with metastatic colorectal cancer who were treated with anti-epidermal growth factor receptor (EGFR) agents. The initial report of this association between KRAS gene mutation and reduced response to anti-EGFR compounds was published by Arrington et al. Furthermore, their retrospective analysis highlighted that patients with wild-type *KRAS* exhibited improved overall survival compared with those with mutant *KRA*S.^11^

CtDNA comprises genetic material released into the bloodstream because of tumor cell necrosis, while cell-free DNA originates from non-cancerous cells and lacks pathological molecular alterations.^12^ Examination of KRAS mutations within ctDNA is an invaluable strategy for cancer detection and surveillance, providing essential insights into tumor genetics and treatment efficacy. Recent studies have suggested that ctDNA analysis could be a promising avenue to identify the K-Ras oncogene in colorectal and gastric cancer.^13^

The members of this protein family are located in the intracellular regions of cell membranes and play a crucial function in signaling via G proteins. When EGFR is activated by its ligand, a conversion of KRAS proteins from the GDP into the GTP form is triggered, resulting in an elevated BRAF concentration in the plasma membrane. BRAF, in turn, activates the MAPK signaling pathway, initiating the expression of proteins crucial for multiple vital pathways, including proliferation, differentiation, survival, angiogenesis, and cell migration. When the KRAS protein is mutated, it remains in its active form, causing uncontrolled cell proliferation and ultimately leading to malignancy.^14^

In 2022, nearly 20 million new cancer cases were reported, and 9.7 million people lost their lives because of cancer. Estimates indicate that approximately one in five people will develop cancer during their lifetime, while roughly one in nine men and one in 12 women will die from the disease. Gastric cancer accounted for 4.9% of diagnosed cases, while colorectal cancer accounted for 9.6%. Moreover, while accounting for an estimated 18.7% of cancer-related deaths, colorectal cancer (9.3%) and gastric cancer (6.8%) are among the leading causes of cancer mortality.^15^

In this study, we assessed the profile of HPV and EBV infections with regard to the prevalence of Ras gene mutations. HPV infection was found in 5.4% of gastric cancer cases. Furthermore, for colorectal cancer, HPV infection was observed in 7.3% of the cancer group and 3.6% of the control group. In a separate study, detection of HPV was recognized as a heightened risk determinant for the development of colorectal cancer.^16^

Moreover, multiple meta-analyses have shown associations between the high-risk HPV types 16, 18, 31, 33, and 35 and an increased risk of colorectal cancer.^17^ It is widely recognized that early proteins of high-risk HPV, including the E5, E6, and E7 oncoproteins, promote cellular alterations, likely contributing to HPV-induced carcinogenesis.^18^ The notable prevalence of high-risk HPV infection in colorectal tumors, along with evidence of viral genome integration, strongly suggests that HPV could play a role in the development of colorectal cancer.^19^

While the precise impact of HPV on colorectal cancer risk has been extensively studied, controversy remains surrounding the association between HPV and colorectal cancer. Multiple publications have proposed that HPV infection within the colonic mucosa could potentially play a role in the development of colorectal cancer.^20,21^

Nevertheless, numerous investigations have failed to definitively establish a connection between HPV and colorectal cancer. In one study, infection of the normal colon mucosa with HPV-18 was identified as a potential risk factor in the development of colorectal cancer.^22^ However, this finding was not corroborated by a subsequent study that predominantly highlighted the importance of HPV-16. The presence of HPV-16 DNA was detected in 21.9% of colorectal adenocarcinomas, contrasting notably with non-adenocarcinomatous tumor samples, where only 3.1% exhibited HPV-16 DNA. Additionally, no evidence of HPV-18 infection was observed in the investigated cases.^23^

The findings of these two studies diverge and differ from our results. In a study conducted by Santos et al., HPV was detected in 27.1% of samples obtained from patients with colorectal carcinoma. Conversely, among NAT obtained from patients, 9.4% of samples tested positive for HPV, showing a significant difference between the two groups (p < 0.0001).^24^ These results stand in contrast to our own findings. Such discrepancies might be attributed to geographical variations in the study populations, regional disparities in HPV prevalence, or differences in the methods employed for HPV detection and the selection of the material for analysis (fresh/paraffin).^25^

In gastric cancer, approximately 9% of patients exhibit EBV within the tumor cells,^26^ although its impact on carcinogenesis and gastric cancer development remains uncertain. Certain studies have suggested the potential existence of a correlation between the onset of gastric cancer and HPV infection, similar to the relationship established with EBV.^27^ Based on a meta-analysis carried out by Zeng et al., a pooled HPV prevalence of 28% was estimated for 1917 cases of gastric cancer from 30 included studies. This finding suggests that HPV prevalence is a significant risk factor in the development of gastric cancer.^28^

The results of our study are in close agreement with those of Fakhraei et al., who revealed that 5% of the samples tested positive for HPV DNA. Nevertheless, it is crucial to emphasize that no statistically significant correlation was observed between gastric cancer and HPV infection in the northern region of Iran.^29^

Based on our findings, we observed that among gastric cancer patients, 51.4% and 13.5% were positive for EBV infection in the tumor and control groups, respectively. In the colorectal group, EBV infections were detected in 49.1% and 16.4% of the cancer and control groups, respectively. Furthermore, there was a statistically significant correlation between EBV infection and both colorectal and gastric cancer (p < 0.001).

Several comprehensive association studies have indicated a potential link between EBV infection and colorectal cancer. These findings are derived from data analysis involving gene expression profiling, protein–protein interactions, and transcriptional and post-transcriptional regulation data. However, it is worth mentioning that the current scientific evidence regarding the presence and role of EBV in colorectal cancer remains insufficient and contradictory.^30^

Maskouni et al. conducted a meta-analysis that included 22 studies and determined that the pooled prevalence of EBV among 1954 colorectal cancer patients was 18%. Additionally, South America exhibits the maximum prevalence of EBV at 30%, while Africa has the lowest prevalence at 0%, as determined by the examination of various geographical regions.^31^ In a separate study, EBV DNA was detected in 60% of tumor samples, and 13.3% of cancer patients had EBV DNA detected in both the tumor and their NAT.^32^

EBV may exert indirect effects on colorectal cancer development by potentially influencing the immune response and promoting inflammation, both well-established factors in cancer progression. In this context, EBV can infect the epithelial cells lining the gastric mucosa, leading to various cellular alterations, including genetic and epigenetic alterations within the infected cells, which can contribute to cancer development. Notably, EBV-associated gastric cancer (EBVaGC) is a prevalent malignant tumor associated with EBV infection. The molecular classification of gastric carcinoma highlights EBVaGC as a distinct cancer subtype with unique oncogenic mechanisms and molecular characteristics.^33^

Shinozaki-Ushiku et al. demonstrated that EBVaGC represents a separate subtype, accounting for nearly 10% of gastric carcinomas.^34^ Tokunaga et al. reported the detection of EBV in 6.7% of gastric cancer cases,^35^ a finding consistent with another study conducted in Iran, which reported a similar percentage of 6.66% of EBV-positive cases among gastric cancers.^36^ In contrast to previous findings, our study revealed varying rates of HPV and EBV coinfection in different types of cancer. Specifically, we observed coinfection rates of 5.4% in gastric cancer, 1.9% in colorectal cancer, and 3.6% in the colorectal control group. In accordance with our findings, Gupta et al. demonstrated coinfection of HPV and EBV in 16% of the sample population, but this coinfection did not show a significant association with clinicopathological variables.^37^ The authors concluded that HPV is highly prevalent in colorectal cancer samples, whereas EBV positivity remains relatively low. Plasma ctDNA levels often increase as the disease progresses, and only 47% of patients in the early stages of cancer have measurable amounts of ctDNA. Notably, in colorectal cancer patients, the *KRAS* codon 12 mutation rate in cell-free DNA is significantly higher than that in healthy subjects.^38^ Our research concerning the evaluation of RAS gene status revealed a statistically significant association (p = 0.01) between KRAS status and gastric cancer. Additionally, we observed a significant (p = 0.03) association between KRAS status and colorectal cancer. Yang et al. demonstrated that *KRAS* mutations were present in 44.2% of colorectal cancers and 37.1% of rectal cancers and were not present in gastric cancers.^39^ Similarly, in a study by Koulouridi et al., KRAS mutations were found in 93.2% of patients, with the *KRAS* G12D subtype (30.1%) being the most prevalent.^40^

In a study that included 200 patients, Hayama et al. revealed that the most prevalent KRAS mutation subtype was G12D. This subtype was observed in 37% of patients who had KRAS mutations, which is consistent with our findings.^41^ KRAS mutations are typically found in approximately 40% of colorectal cancer patients (stages II–IV), and their established role as adverse predictive factors for the effectiveness of anti-EGFR therapy has been confirmed.^42^ In the present study, G12D mutations were observed in 54% of patients with gastric cancer and in 35% of patients with colorectal cancer among ctDNA samples.

High-risk HPV infections and mutational activation of the KRAS gene are frequent occurrences in the context of colorectal carcinogenesis. No significant relationship has been identified between HPV positivity and KRAS mutations despite an examination of the association between these two markers.^43^ The KRAS G12D mutation appears to hinder an effective immune response against tumors.^44^ Numerous trials, both in preclinical and clinical settings, have sought therapeutic options for KRAS-mutated patients with colorectal cancer. In translational oncology, innovative strategies are emerging to combat colorectal carcinoma. These include targeting KRAS G12D for adoptive T-cell transfer, exploring KRAS vaccines, and investigating molecules that target KRAS mutation pathways.^45^

KRAS mutations have been established as crucial predictive factors, although their prognostic relevance is currently being assessed. Numerous studies have demonstrated that colorectal cancer patients with KRAS mutations experience a poorer prognosis.^46^ He et al. found that colorectal cancer patients harboring KRAS-G12D mutations and presenting synchronous metastasis manifest a disease phenotype associated with potentially worse prognostic outcomes.^47^ Based on prior research and our findings, the identification of KRAS mutations, particularly the G12D subtype, holds significant importance for colorectal cancer patients because of their prognostic value and potential therapeutic implications.

Further investigations should be pursued to thoroughly explore this hypothesis. Additionally, it is worth noting that KRAS mutations have also been established in gastric cancer, and alterations in the RAS pathway, caused by both RAS and BRAF mutations, play a role in the pathogenesis of gastric cancer.^48^

KRAS mutations have indeed emerged as significant predictive markers. In our study, within EBV-positive gastric cancer samples, we noted 8.1% exhibited wild-type characteristics and 43% displayed heterozygous mutations, whereas in the control group, the percentage of heterozygous mutations was 14.6% (p < 0.001). The frequency of KRAS mutations in gastric cancer was reported as 6.67%.^49^ In contrast, for colorectal cancer, 1.8% of EBV-positive samples demonstrated wild-type status and 47.2% were heterozygous mutations, in contrast to the control group, which exhibited 14.7% heterozygous mutations (p < 0.01).

This finding suggests that KRAS mutations might have greater importance in colorectal cancer than in gastric cancer. Consistently, concerning colorectal cancer patients with HPV-positive samples, we noted 1.8% wild-type specimens, 5.4% that displayed heterozygous mutations, and 3.2% presenting homozygous mutations. In a separate study, 56% of HPV-positive tumors carried the KRAS mutation.^50^ This finding suggests that the combination of high-risk HPV infections and mutational activation of the KRAS gene are common occurrences in the process of colorectal carcinogenesis.

It is reasonable to infer that KRAS mutational activation plays a prevalent role in colon tumorigenesis, and HPV infection may serve as an additional factor to promote malignant transformation.^50^ The development of targeted therapies that are specifically designed for malignancies with KRAS mutations is the primary focus of ongoing efforts. Additionally, the integration of genetic and viral factors has the potential to provide valuable insights into the initiation and progression of this form of cancer.

In this study, control group plasma was also examined for the KRAS gene for comparison with the cancer group plasma. The results showed a significant difference between the control and cancer groups, indicating a difference in the KRAS gene pattern in gastric and colorectal cancer. In addition, the results regarding KRAS gene status in colorectal and gastric cancer aligned with the Hardy–Weinberg equilibrium. This finding suggests that the genetic balance of heterozygous and homozygous status is maintained within the population. It can be inferred that the identified mutations were not solely instigated by cancer; rather, they likely originated in preceding generations. It is plausible that individuals carrying a mutant gene are more predisposed to cancer development than those with the wild-type gene.

The limitation of our study is related to the sample size because examining a large number of cancer patients for the KRAS gene, HPV, and EBV infections could provide results that are generalizable to the population.

## Conclusion

In this study, we explored the relationship between specific viruses and the development of GI cancers, particularly gastric and colorectal cancers. Although these associations have drawn considerable attention in recent years, the detailed mechanisms by which these viruses contribute to cancer development remain insufficiently understood.

Further research is necessary to elucidate the role of HPV and EBV in the pathogenesis of colorectal and gastric malignancies to fully comprehend the molecular mechanisms driving carcinogenesis. KRAS mutations, which are among the most frequently observed genetic alterations in colorectal and gastric cancers, are consistently found in tumor samples.

Given their pivotal role in tumor initiation and progression, KRAS mutation targeting represents a promising therapeutic strategy. A deeper understanding of the tumorigenic and metastatic processes in these cancers may reveal critical therapeutic opportunities. Consequently, evaluation of the KRAS mutational status is essential to guide treatment strategies for both colorectal and gastric cancer management.
